# Pattern of humoral immune response to *Plasmodium falciparum *blood stages in individuals presenting different clinical expressions of malaria

**DOI:** 10.1186/1475-2875-7-186

**Published:** 2008-09-24

**Authors:** Fabiana MS Leoratti, Rui R Durlacher, Marcus VG Lacerda, Maria G Alecrim, Antonio W Ferreira, Maria CA Sanchez, Sandra L Moraes

**Affiliations:** 1 Institute of Tropical Medicine of São Paulo, University of São Paulo, Av. Dr. Enéas de Carvalho Aguiar, 470, 05403-000, São Paulo, Brazil; 2 Foundation of Tropical Medicine of Manaus, Amazonas, Brazil

## Abstract

**Background:**

The development of protective immunity against malaria is slow and to be maintained, it requires exposure to multiple antigenic variants of malaria parasites and age-associated maturation of the immune system. Evidence that the protective immunity is associated with different classes and subclasses of antibodies reveals the importance of considering the quality of the response. In this study, we have evaluated the humoral immune response against *Plasmodium falciparum *blood stages of individuals naturally exposed to malaria who live in endemic areas of Brazil in order to assess the prevalence of different specific isotypes and their association with different malaria clinical expressions.

**Methods:**

Different isotypes against *P. falciparum *blood stages, IgG, IgG1, IgG2, IgG3, IgG4, IgM, IgE and IgA, were determined by ELISA. The results were based on the analysis of different clinical expressions of malaria (complicated, uncomplicated and asymptomatic) and factors related to prior malaria exposure such as age and the number of previous clinical malaria attacks. The occurrence of the H131 polymorphism of the FcγIIA receptor was also investigated in part of the studied population.

**Results:**

The highest levels of IgG, IgG1, IgG2 and IgG3 antibodies were observed in individuals with asymptomatic and uncomplicated malaria, while highest levels of IgG4, IgE and IgM antibodies were predominant among individuals with complicated malaria. Individuals reporting more than five previous clinical malaria attacks presented a predominance of IgG1, IgG2 and IgG3 antibodies, while IgM, IgA and IgE antibodies predominated among individuals reporting five or less previous clinical malaria attacks. Among individuals with uncomplicated and asymptomatic malaria, there was a predominance of high-avidity IgG, IgG1, IgG2 antibodies and low-avidity IgG3 antibodies. The H131 polymorphism was found in 44.4% of the individuals, and the highest IgG2 levels were observed among asymptomatic individuals with this allele, suggesting the protective role of IgG2 in this population.

**Conclusion:**

Together, the results suggest a differential regulation in the anti-*P. falciparum *antibody pattern in different clinical expressions of malaria and showed that even in unstable transmission areas, protective immunity against malaria can be observed, when the appropriated antibodies are produced.

## Background

The acquisition of natural immunity to malaria is slow and requires repeated parasite exposure to be maintained [1]. This immunity reduces the risk of both severe and mild malaria but does not suppress parasitaemia. The defense mechanisms require cooperation among antibodies (Ab) and cellular immune responses [2]. There is evidence that (Ab)-dependent mechanisms play an important role in the reduction of parasitaemia and can diminish clinical symptoms in humans, as demonstrated by the passive transfer of hyperimmune immunoglobulin G (IgG) [3,4].

Cooperation between monocytes and Ab appears to be crucial in acquired protective immunity [5]. The Ab produced in response to infection are of particular importance, since certain isotypes known as cytophilic Ab can cooperate with monocytes via FcγRI and FcγRII receptors in opsonization and phagocytosis or participate in both antibody-dependent cellular inhibition (ADCI) as well as antibody-dependent cellular cytotoxicity (ADCC) [6-8]. Protection against the blood stage of *Plasmodium falciparum *seems to depend on the proportion of specific cytophilic Ab relative to the proportion of non-cytophilic Ab [9]. The predominance of IgG_1 _and IgG_3 _cytophilic Ab in endemic areas has been associated with either lower parasitaemia [10] or a lower risk of malaria attack [11,12]. Otherwise, noncytophilic Ab, such as IgG_4_, may inhibit effector mechanisms by competing with cytophilic Ab and are considered nonprotective [7,13]. IgG_2 _is non-cytophilic, but could be correlated with protection in individuals carrying a specific allelic variant of monocytes FcγRIIA receptor that can bind IgG_2 _[14].

IgE levels and IgE anti-plasmodial Ab are elevated in human and experimental malaria infections, but their role in protection and/or pathogenesis is not well-established in malaria [15-17]. In fact, a negative correlation between the IgE level and placental parasitaemia [18,19] and levels of haemoglobin and platelets [20] has been found. The IgE level was higher in cerebral *P. falciparum *malaria than in uncomplicated malaria [16,21], while among patients with severe malaria, the increase in IgE levels was related to the deepness of the coma [20]. Recent data showed higher IgE functional activity in asymptomatic and uncomplicated malaria patients than in severe or cerebral malaria groups [22].

Contradictory results have been also found in relation to IgM Ab. Even though there are indirect epidemiological data suggesting that IgM Ab does not participate in protection [23,24], some evidence suggests a protective role in cases such as: (a) mice with an X-linked recessive B-cell deficiency do not produce IgM and are susceptible to *Plasmodium yoelii *[24]; (b) the addition of monoclonal IgM Ab to a malaria vaccine, raising its protective properties [25]; (c) the IgM level, which correlated with a decrease of the parasitaemia in individuals living in an area of hyperendemic malaria [26]. In terms of IgA Ab, no specific function in malaria has been ascribed to these antibodies to date.

The gradual development of natural immunity to malaria has been observed in areas where malaria transmission is high and stable. The great majority of the studies on the prevalence of different immunoglobulin isotypes and their role in malaria infection has been conducted in such areas, but little is known about areas with unstable malaria, such as the Amazon Basin of Brazil. The main objective of this work was to compare the humoral immune response in three groups of *Plasmodium*-infected subjects living in malaria endemic areas of Brazil: (a) subjects with non-cerebral complicated malaria, (b) subjects with uncomplicated malaria, and (c) asymptomatic subjects – investigating the distribution of *P. falciparum *blood stages-specific isotypic Ab IgG, IgG1, IgG2, IgG3, IgG4 IgM, IgE and IgA. The avidity of IgG subclasses, a parameter of humoral immune response quality that has received little attention in studies on malaria, was also evaluated in this study.

## Materials and methods

### Study area, subjects and serum samples

Venous blood samples were collected from 233 residents from two cities, Peixoto de Azevedo and Manaus, in the Amazon Basin of Brazil. Peixoto de Azevedo is in the state of Mato Grosso (PA/MT) and Manaus is the capital of the state of Amazonas (MA/AM). The equatorial and humid climate of both cities is characterized by a rainy season from November to April and a dry season from May to October. Malaria transmission is unstable, with an increase at the end of the rainy season. Informed consent was obtained individually from all participants before the blood sample was taken. This study was approved by the Committee of Ethics of the Faculty of Medicine of the University of São Paulo, of the Institute of Tropical Medicine of São Paulo (IMT/SP) and of the Foundation of Tropical Medicine of Amazonas (FMT/AM). A questionnaire containing vital statistics and reports of the number of previous malaria attacks was available from all individuals.

At the time of blood collection, thick blood films and/or QBC^® ^(Quantitative Buffy Coat) were prepared according to standard protocols for all subjects. Based on the clinical expression of malaria, three groups were defined: (a) 70 subjects presenting non-cerebral complicated malaria (52 *P. falciparum *and 18 *Plasmodium vivax*) (CM group); (b) 148 subjects presenting uncomplicated malaria (92 *P. falciparum*, 32 *P. vivax*, 23 *P. falciparum *and *P. vivax*, and 1 *Plasmodium malariae*) (UM group), and (c) 15 subjects with *Plasmodium *infection, but without malaria symptoms (9 *P. falciparum*, 1 *P. vivax*, 4 *P. falciparum *and *P. vivax*, and 1 *P. malariae*) (AS group). Non-cerebral complicated malaria was defined as either severe anaemia and/or high parasitaemia, hypoglycaemia < 40 mg/dL, serum creatinine level > 1,5 mg/dL. Uncomplicated malaria was characterized by a positive blood smear and fever without other causes of infections and no manifestations of severe malaria as described above. Ninety serum samples from malaria-naïve blood donors were used as negative control in the assays. Table 1 shows some characteristics of the population.

**Table 1:** Characteristics of the studied population.

Characteristics	Clinical expressions of malaria			Total
	CM (n = 70)	UM (n = 148)	AS (n = 15)	n = 233
median age in years (25%–75%)	25.8 (17.0–40.0)	28.0 (22.0–37.0)	30.0 (25.8–39.5)	30.2
Sex (male/female %)	48.4/51.6	82.4/17.6	86.7/13.3	
mean number of previous malaria attacks	3.7 ± 6.5	11.9 ± 8.9	14.8 ± 8.3	10.2

CM = complicated malaria, UM = uncomplicated malaria, AS = asymptomatic infection

### Enzyme-linked immunosorbent assay (ELISA) for IgG subclasses and IgE Ab

IgG subclasses and IgE Ab were detected with ELISA using a Zwittergent-extracted *P. falciparum *blood stages antigen as described above [27]. Flat-bottom plastic microplates (Polysorb, NUNC) were coated with a *P. falciparum *antigen diluted to 5 μg/mL in phosphate buffered saline (PBS) (50 μL/well) for 2 h at 37°C and subsequently for 18 h at 4°C in a moist chamber. Plates were saturated with 5% skim milk in PBS-Tw20 (200 μL/well) for 1 h at 37°C. Serum dilutions in 1% skim milk in PBS-Tw20 (serum diluent) (50 μL/well) were incubated for 40 min at 37°C in duplicate wells, 1:50 for IgG1, IgG2 and IgG3, 1:5 for IgG4. For IgE detection, IgG Abs were first depleted by treating them with a 1:20 serum dilution with a sheep anti-human IgG (Rf-absorbent, Dade Behring) (1:1). The biotinylated sheep anti-human IgE antibody (Sigma Chem. Co.) was diluted to 1:500 in serum diluent (50 μL/well) and biotinylated mouse monoclonal Ab specific to each IgG isotype were used at the following dilutions in serum diluent (50 μL/well): anti-IgG1 (clone 8c/6-39; Sigma Chem. Co.), 1:1,000; anti-IgG2 (clone HP 6014; Sigma Chem. Co.), 1:500; anti-IgG3 (clone HP 6050; Sigma Chem. Co.), 1:500; anti-IgG4 (clone HP 6025; Sigma Chem. Co.), 1:500. The peroxidase-labeled streptavidin was diluted to 1:1,000 (50 μL/well) in serum diluent and incubated for 40 min at 37°C. The enzymatic reaction was developed with hydrogen peroxide and tetramethylbenzidin (Sigma Chem. Co.) diluted in citrate-phosphate buffer (H_2_O_2_-TMB) (50 μL/well) for 30 minutes at 37°C, and the reaction was stopped with 25 μL/well of 2N H_2_SO_4_. Spectrophotometric reading at 450 nm was performed on a Titertek Multiskan (MKII)-MCC/340 spectrophotometer.

The results were expressed as a reactivity index (RI) or concentration. The reactivity index (RI) was calculated as the ratio of the serum sample optical density to the cut-off optical density. Serum samples with an RI equal to or greater than 1.0 were considered positive. The cut-off was defined as the mean plus 2 SD of the optical density values obtained with 90 negative control sera. Standard curves were drawn by testing in each microplate six serial dilutions (5120, 1280, 320, 80, 20 and 5 ng/mL) of myeloma protein of any the four IgG subclasses (Sigma Chem. Co.) or IgE myeloma. Coating was done as described above with 50 μL/well of a mixture (1:1) of one κ and one λ myeloma protein of each isotype. Concentrations of antigen-bound Ig isotypes were calculated from standard curves using a four-parameter log-logit function, with the aid of the ELISA programme developed by Plikyatis *et al *[28]. Competition experiments with IgG1, IgG2, IgG3 and IgG4 purified myeloma (Sigma Chem. Co.) were performed to check the specificity of anti-human IgG subclasses and no cross-reaction was observed.

The avidity of anti-*P. falciparum *IgG and IgG subclasses was assessed with an urea-elution based ELISA. Microplates were coated and saturated as described above. After serum samples were incubated, 8 M urea in PBS was added (100 μL/well) for 5 min to one of the duplicates, while PBS-Tw20 was added to the other duplicate. After washing, the assay was completed as described above. The avidity index (IA) was calculated as the ratio of the urea-treated serum sample optical density to the non-treated serum sample optical density, and multiplied by 100. All values higher than 50% were ranked as high avidity and values equal or lower than 50% were considered low avidity.

### ELISA for IgG, IgM and IgA Ab

Slight differences were noted among anti-*P. falciparum *IgG, IgM and IgA Ab when analysed as described above. Serum dilutions in serum diluent (50 μL/well) were incubated for 40 min at 37°C in duplicate, 1:100 for IgG and IgM, and 1:25 for IgA. The peroxidase-labeled sheep anti-human IgG, IgM and IgA were diluted to 1:10,000, 1:20,000 and 1:2,000, respectively, in serum diluent (50 μL/well) and incubated for 40 min at 37°C. The enzymatic reaction was developed with H_2_O_2_-TMB (50 μL/well) for 30 minutes at 37°C, and the reaction was stopped with 25 μL/well of 2N H_2_SO_4_. A spectrophotometric reading at 450 nm was performed on a Titertek Multiskan (MKII)-MCC/340 spectrophotometer.

### Total human IgE and IgA measurement

The concentrations of total human IgE and IgA were determined in 66 serum samples from malaria endemic areas using commercial nephelometry kits (Dade-Behring, Germany).

### Determination of FcγRIIA H/R131 polymorphism

FcγRIIA H/R131 polymorphism was determined in 126 DNA samples from the subjects by using an allele specific restriction enzyme digestion method [29]. DNA extraction was performed as described by Snounou *et al *[30] and modified by Ferreira *et al *[31]. First, the FcγRIIA-H/R gene was amplified by PCR and the resulting product was digested by the *Bsh1236I *(FnuDII) enzyme. The H131 allele contains a *Bst*UI site in the 3' region and the R131 allele contains two *Bst*UI sites in the 3' and 5' regions. After *Bst*UI digestion, the H/H131 genotype produces a 343 fragment, the R/R131 genotype produces a 322 fragment and the H/R131 genotype produces one of each fragment.

### Statistical analysis

Analysis was carried out using SigmaStat software (Jandel Scientific Corporation, CA). Antibody frequencies among the three malaria groups were assessed by a χ^2^-test. Antibody levels in the three malaria groups were compared using the non-parametric Kruskal-Wallis (KW) test and the Mann-Whitney (MW) test when two groups were compared. Correlations were assessed by Spearman rank test (Sp), using real or logarithmically transformed values. *P *values ≤ *0.05 *were considered significant.

## Results

### Pattern of isotype response against *P. falciparum *blood stages considering different clinical expressions of malaria

IgG, IgG1, IgG2, IgG3, IgG4, IgM, IgA and IgE isotypes levels were tested in *P. falciparum *blood-stage antigens of 233 *Plasmodium*-infected subjects presenting different clinical expressions of malaria: complicated malaria (CM), uncomplicated malaria (UM) and asymptomatic malaria (AS). The results are presented on Table 2. Index of avidity of IgG, IgG1, IgG2, IgG3 and IgG4 are also shown.

**Table 2:** Results of the levels of different antibodies isotypes and their avidity against *P. falciparum *blood stages determined by ELISA in serum from 233 individuals infected with *Plasmodium *and classified according to the different clinical expressions of malaria: complicated malaria (CM), uncomplicated malaria (UM) and asymptomatic malaria (AS).

	Levels of antibodies against *P. falciparum *blood stages			Statistical
		
	CM	UM	AS	*P*
IgG1*	2.28 (0.80–10.37)	18.40 (7.23–44.8)	38.4 (3.95–71.8)	< 0.0001
AI-IgG1+	35.1 (24.7–64.7)	66.4 (44.4–86.7)	71.9 (62.2–84.6)	< 0.0001
IgG2*	1.33 (0.67–3.00)	3.92 (2.26–17.1)	9.81 (2.25–89.8)	< 0.0001
AI-IgG2+	20.4 (16.8–68.6)	58.0 (37.5–76.6)	75.3 (62.0–92.6)	0.014
IgG3*	0.43 (0.19–0.91)	1.53 (0.72–3.74)	2.41 (0.53–9.47)	< 0.0001
AI-IgG3+	43.5 (22.0–53.6)	42.9 (27.1–66.5)	37.1 (29.4–53.3)	0.813
IgG4*	0.06 (0.00–0.14)	0.01 (0.00–0.04)	0.01 (0.00–0.03)	0.002
AI-IgG4+	40.0 (2.8–77.8)	48.9 (36.0–59.1)	-	0.471
IgG#	2.47 (0.73–5.44)	2.92 (1.36–4.39)	4.71 (1.59–6.58)	0.190
AI-IgG+	48.4 (34.7–69.7)	63.3 (46.8–73.2)	63.4 (58.2–69.8)	0.036
IgE*	0.07 (0.03–0.16)	0.03 (0.00–0.11)	0.03 (0.01–0.21)	0.07
IgM#	1.08 (0.65–2.08)	0.86 (0.59–1.50)	1.04 (0.58–2.00)	0.106
IgA#	0.93 (0.56–2.09)	0.87 (0.60–1.88)	-	0.661

*Levels expressed as concentration median (μg/mL)

# Levels expressed as the index of reactivity (IR) calculated as [sample optical density (OD)/cut-off OD]

+ AI = avidity index mean, calculated as (urea-treated sample OD/PBS sample OD)*100

*P *value for Kruskal Wallis non-parametric test

Levels of anti-*P. falciparum *IgG1, IgG2 and IgG3 Ab were higher in the UM and AS groups than in the CM group (KW, *P *< 0.0001). In contrast, levels of IgG4 were higher in the CM group than in the UM and AS groups (*P *= 0.002). Also, IgE levels were higher in CM but without significant differences (KW, *P *= 0.07). Levels of IgG, IgM and IgA Ab did not differ among the groups (KW, *P *> 0.05). Higher avidity indexes were observed for IgG, IgG1 and IgG2 antibodies in the UM and AS groups compared to the CM group (KW, *P *= 0.036; *P *< 0.0001; *P *= 0.014, respectively) (Table 2).

The prevalence of IgG1, IgG2 and IgG3 Ab was significantly higher in the UM and AS groups than in the CM group (χ^2 ^= 42.5%; *P *< 0.0001; χ^2 ^= 12.7%; *P *= 0.002; χ^2 ^= 10.1%; *P *= 0.006, respectively) (Table 3). The prevalence of IgG4, IgE, IgG, IgM and IgA Ab did not differ significantly among the three groups (*P *> 0.05). High-avidity IgG, IgG1, IgG2 and IgG4 Ab predominated in the UM and AS groups, while low-avidity Ab predominated in the CM group, although a significant difference was only noted for IgG1 Ab (χ^2 ^= 16.4; *P *= 0.0003). Low-avidity IgG3 Ab predominated in the three groups, but with no significant differences (*P *> 0.05) (Table 3).

**Table 3:** Frequency of antibodies against *P. falciparum *blood stages determined with ELISA in serum from 233 individuals infected with *Plasmodium *and classified according to the different clinical expressions of malaria: complicated malaria (CM), uncomplicated malaria (UM) and asymptomatic malaria (AS).

	Frequency of individuals (%)					
	
Isotype	CM		UM		AS	
			
	total	high/low avidity	total	high/low avidity	total	high/low avidity
IgG1	50.0	58.8/41.2	89.9	86.4/13.6	73.3	100.0/0
IgG2	24.3	47.1/52.9	47.3	85.5/14.5	60.0	100.0/0
IgG3	41.4	65.5/34.5	64.2	73.1//26.9	60.0	66.7/33.3
IgG4	18.0	55.6/44.4	16.5	77.3/22.7	6.7	100.0/0
IgG	71.4	81.4/18.6	79.1	93.8/6.2	73.3	100.0/0
IgE	39.1	-	37.6	-	35.7	-
IgM	58.6	-	39.9	-	53.3	-
IgA	46.2	-	40.0	-	60.0	-

With regards to the *Plasmodium *species, there was a significant difference in IgG1 Ab frequency when *P. falciparum *(78.4%) was compared with *P. vivax *(62.7%) infected patients (χ^2 ^= 4.16; *P *= 0.04); and when *P. vivax *(62.7%) was compared with *P. falciparum *and *P. vivax *(92.6%) infected patients (χ^2 ^= 6.55; *P *= 0.01). On the other hand, no significant difference was observed for all other Ab classes and subclasses studied (*P *> 0.05).

Different immunoglobulin isotypes can react with the same epitopes but can influence the course of an infection differently. In view of this fact, some of the interactions of different isotypes against *P. falciparum *were compared in the groups of subjects presenting different clinical expressions of malaria. Interactions of IgG1, IgG2 and IgG3 were higher among the UM and AS groups, while interactions of IgG4 and IgE were higher in the CM group. Higher IgG2/IgG4 and IgG/IgM ratios were observed in the UM and AS groups (Table 4).

**Table 4:** Comparison of the interactions of isotypes against *P. falciparum *blood stages levels of 233 *Plasmodium*-infected subjects presenting different clinical expression of malaria.

	Clinical expression of malaria			
		
Interaction	Complicated	Uncomplicated	Asymptomatic	(P)*
IgG1+IgG3/IgG2+IgG4	2,16	3,35	1,70	0,061
IgG1+IgG2+IgG3/IgG4	0,36	9,12	32,1	< 0,001
IgG1+IgG3/2	1,45	10,94	23,94	< 0,0001
IgE+IgG4	0,16	0,06	0,06	0,0100
IgG2/IgG4	12,2	154,1	482,7	< 0,0001
IgG3/IgG4	4,29	39,79	82,83	< 0,0001
IgE+IgG4/IgG2	0,106	0,008	0,01	< 0,0001
IgM/IgG	0,565	0,39	0,36	0,032
IgG/IgM	1,77	2,56	2,81	0,031
IgG1+IgG2+IgG3/IgM+IgE+IgG4	5,32	13,87	18,87	< 0,0001

*P values were determined by the non-parametric Kruskal Wallis test.

### Association with variables indicative of prior malaria exposure

The influence of age and the number of previous clinical malaria attacks on the levels of the isotypes specific for *P. falciparum *blood-stage antigens was evaluated. Age was positively correlated with IgG (Sp, r = 0.13; *P *= 0.033), IgG1 (Sp, r = 0.17; *P *= 0.008) and IgG2 Ab (Sp, r = 0,16; *P *= 0.008). The number of previous clinical malaria attacks was positively correlated with IgG, IgG1 (Sp, r = 0.51; *P *= 0.00), IgG2 (Sp, r = 0.43; *P *= 0.00) and IgG3 (Sp, r = 0.33; *P *= 0.00), and negatively correlated with IgG4 (Sp, r = -0.23; *P *= 0.001). Levels of IgG1, IgG2, IgG3 and IgG were significantly higher in subjects reporting more than five previous clinical malaria attacks than among those reporting one to five previous clinical malaria attacks or those undergoing their first malaria episode (KW, *P *< 0.0001). Conversely, the levels of IgG4 and IgE were significantly higher in subjects undergoing their first malaria episode (KW, *P *= 0.0004). No significant difference was observed in the levels of IgM and IgA Ab (*P *> 0.05).

The mean avidity indexes (mAI) of the IgG1 Ab increased significantly with the number of previous clinical malaria attacks: above five previous clinical malaria attacks (mAI = 71.7%), one to five previous clinical malaria attack (mAI = 52.2%) and the first malaria episode (mAI = 22.9%) (KW, *P *< 0.0001). The AI of IgG, IgG2, IgG3 and IgG4 Ab were not statistically different (KW, *P *> 0.05).

### Determination of total IgE and IgA

Total IgE and IgA levels were investigated in 66 serum samples of the *Plasmodium*-infected subjects using a nephelometry assay. The median of the total IgE levels was 1,095 UI/mL, which is higher than normal human levels (100 UI/mL); only four subjects had normal total IgE levels. Of the 62 serum samples with high total IgE levels, 40 (64.5%) had anti-*P. falciparum *IgE Ab and 22 (35.5%) did not (*P *= 0.002). The median of the total IgA levels was 2.00 g/L, while normal levels are between 0.7 and 2.0 g/L. Three serum samples showed that total IgA levels were higher than normal, while four serum samples were below normal levels.

### Determination of FcγRIIA H/R131 polymorphism

Since the occurrence of FcγRIIA H/R131 polymorphism grants IgG2 Ab a cytophilic function and results show higher IgG2 levels in uncomplicated and asymptomatic malaria than in complicated malaria, the distribution of FcγRIIA H/R131 in the population was analysed. The FcγRIIA H/R131 genotype was examined using genomic DNA from 126 *Plasmodium*-infected subjects. Overall, the H131 allele was found in 44.4% of the subjects; 15.1% were H/H, 58.7% were H/R and 26.2% were R/R. IgG2 Ab levels were higher in the asymptomatic subjects with the H131 allele (78.6 mg/mL) than in those without it (38.1 mg/mL), but this difference was not significant. A significant difference was observed in IgG2 levels when asymptomatic subjects with the H131 allele were compared to symptomatic subjects without the H131 allele (4.5 mg/mL) (MW, *P *= 0.02) (Table 5).

**Table 5:** IgG2 levels against *P. falciparum *blood stage extract detected with ELISA and the FcγRIIA H131 divided into two groups (individuals with or without malaria symptoms) genotype.

Individuals	Allele	IgG2 antibodies		
	FcγRIIA H131	[] *Md*	N pos	% pos
Symptomatic	R/R (n = 29)	4.5 (2.5–12.9)	14	48.3
(n = 113)	H/H or H/R (n = 84)	4.4 (2.6–18.5)	43	51.2
asymptomatic	R/R (n = 4)	5.6 (1.5–23.6)	2	50.0
(n = 13)	H/H or H/R (n = 9)	78.6 (4.9–94.2)	7	77.8

*Md *= median of concentration (μg/mL)

## Discussion

In this study it was investigated how IgG and its four subclasses, IgM, IgE and IgA isotypes, work against *P. falciparum *blood stages of individuals naturally exposed to malaria living in different regions of Brazil. The results of the different classes of immunoglobulins were analysed in relation to the different clinical expressions of malaria: complicated (CM), uncomplicated (UM) and asymptomatic (AS) malaria. Factors related to prior malaria exposure such as age and the number of previous clinical malaria attacks were also analysed.

All the subjects were infected with *P. falciparum *and/or *P. vivax*. It is likely that, due to the fact that a great number of these subjects had suffered multiple malaria episodes in the past and as a result of the cross-recognition of antigens, the frequency of antibodies did not differ significantly among the different infections; in any case, all subjects were analysed.

The IgG, IgG1, IgG2 and IgG3 antibody levels and their respective frequencies of positivity were higher in the UM and AS groups than in the CM group. The isotype levels were positively correlated with the number of past malaria attacks and the IgG, IgG1 and IgG2 antibody levels were positively correlated to age. These results are in line with those of other authors who demonstrated that cytophilic antibodies IgG1 and IgG3 were related to uncomplicated malaria. More specifically, several studies showed that specific IgG1 and IgG3 antibodies could protect against malaria, while lgG4 antibodies probably do not protect against the disease [5,11,12,32-36]. A recent study in Sudan also found lower prevalence of IgG antibodies against three *P. falciparum *merozoite surface protein (MSP) MSP antigens, MSP1-19, MSP2A, and MSP2B in individuals with severe malaria than in uncomplicated [37]. A recent cohort study of children in Burkina Faso has investigated different isotypes against four vaccine candidates antigens, apical membrane antigen 1 (AMA1), MSP1-19, MSP3, and glutamate-rich protein (GLURP). They observed association among IgG, IgG3 and IgG4 against GLURP and a borderline association of IgG to MSP3 with reduced risk of malaria [38].

There have been reports of asymptomatic malaria in these Brazilian regions, suggesting that a certain degree of natural immunity can develop [39,40]. Considering that the development of immunity depends on the degree of previous exposure to the disease, the positive correlation with factors related to prior malaria exposure suggest that these isotypes could play a role in protection against the disease; this idea is supported by the varying frequencies detected among the different clinical expressions of the illness.

In spite of the fact that the IgG2 antibodies did not participate in the mechanisms that caused opsonization, phagocytosis and ADCI as they do not bind to the FcγR receptors (principally, FcγRIIA), the results showed a predominance of IgG2 antibodies among individuals from the UM group. For this, the R131H polymorphism in the FcγRIIA receptor gene was investigated. FcγRIIa is a low-affinity IgG receptor with an important role in neutralizing events of malaria parasites mediated by monocytes [41]. Its ability of binding immunoglobulin subtypes is influenced by a polymorphism within the gene. An amino acid change at position 131 within the FcγRIIa molecule, arginine (R131) or histidine (H131) is critical for the binding of human IgG2 that binds efficiently to FcγIIa-H131 but not to FcγRIIa-R131, although both FcγRIIa allotypes interact with IgG1 and IgG3 [13]. Previous studies have indicated that FcγRIIa polymorphism has an impact on the susceptibility/resistance for malaria, although their results have been contradictory. In some studies, the H/H131 genotype was significantly associated with susceptibility to severe malarial disease [42,43] while in other studies the H/H131 genotype was associated with mild malaria [44] or the H131 alleles were mildly protective against high-density parasitaemia [45]. In relation to FcγRIIa-R/R131 genotype, it has been associated with protection against high levels of *P. falciparum *parasitaemia [45] and against malarial disease [43,46]. In contrast, it was also associated with the development of severe malaria [44]. This discrepancy likely reflects differences in study design or in malaria exposure rates, as well as genetic differences between the populations. FcγRIIa polymorphism plays an important role in other infections. FcγRIIa H/H 131 has been associated to protection from encapsulated bacterial infections, in which IgG2 is critical in host defense [47]. The R/R131 genotype has been found to be a risk factor for recurrent bacterial respiratory infections [48,49].

In the present study, the H131 allele was found in 44.4% of the subjects; 15.1% were H/H, 58.7% were H/R and 26.2% were R/R. The frequency of the H131 allele was very similar to that observed in a population of Burkina Faso (43%) [14], when high levels of IgG2 specific for *P. falciparum *antigens were also associated to protection against malaria. Here, the levels of IgG2 Ab were higher in the asymptomatic subjects with the FcγRIIA H/R131 allele (78.6 mg/mL) than in those without this allele (38.1 mg/mL), but this difference was not significant. However, a significant difference was found in the levels of IgG2 of asymptomatic individuals with the FcγRIIA H/R131 allele and the symptomatic individuals without allele H131 (4.5 mg/mL) (MW, *P *= 0.02). The high levels of IgG2 that were found in AS individuals carrying the H131 polymorphism could suggest that IgG2 plays a role as a cytophilic antibody among this population.

The IgG4 levels showed a negative correlation with the number of previous malaria episodes. In addition, the frequency of positivity and the IgG4 levels were higher among subjects from the CM group. The absence of malaria protection in IgG4 antibodies has been observed by other authors [9,14]. IgG4 inhibits the action of cytophilic antibodies lgG1 and lgG3 *in vitro*; these antibodies are responsible for mediating the opsonization of infected erythrocytes [5]. Aucan *et al *observed that the positive correlation of IgG4 with the risk of infection was stronger when IgG2 levels were low, suggesting that IgG4 could be neutralizing the cellular cytotoxicity mediated by monocytes or other effector cells that depend on IgG2. Therefore, IgG4 could block the protection provided by IgG2 [14].

Another essential aspect of the humoral immune response is the functional affinity or avidity of the antibodies; this is the measurement of the force between the antigen and the antibody and it can reflect the quality of the antibodies involved in protection or in different phases of the illness. When studying the avidity of anti-*P. falciparum *blood stages antibodies among populations from the endemic area with stable transmission in Africa and unstable transmission in the Brazilian Amazon Basin, Ferreira *et al *noted a predominance of high avidity antibodies, particularly IgG1, in the clinically immune African subjects [50]. Among patients from the Brazilian Amazon low avidity antibodies were found during the acute phase of the disease; these were probably the result of the polyclonal activation of B cells. Two months following chemotherapy, the proportion of high avidity IgG antibodies had increased significantly, though the concentration of cytophilic antibodies had decreased. These authors postulated that high-quality anti-*P. falciparum *antibodies could be initially produced during the intra-erythrocytic growth of the parasite, and that these antibodies react when parasitaemias are still low, with higher effectiveness against the severity of the disease. In the current study, low avidity antibodies of all the immunoglobulins predominated among subjects from the CM group, which could reflect the prevalence in this group of individuals with few previous malaria episodes. On the other hand, the predominance of low avidity antibodies could show that uncontrolled infection is related to an expression of immunodeficiency [50]. In general, IgG3 antibodies were low avidity, independently of the clinical expression of malaria. This could be explained by their short half-life (~7–8 days) compared to the half-life of subclasses IgG1, IgG2 and IgG4 (~23 days) [51,52] and it probably suggests that these antibodies are the main subclass of antibodies induced in response to new variants of polymorphic antigens of the erythrocytic stage of *P. falciparum *[53].

High overall levels of IgE have been found in populations exposed to malaria [17], a fact that was also observed in 94% of the individuals studied. However, there is some controversy as to the role of specific IgE antibodies and whether they serve to protect against the disease or to worsen the illness. The following findings could support the theory that specific IgE antibodies provide protection against malaria: a) positive correlations between IgE levels and age [18,19]; b) high concentration of IgE among individuals with severe non-comatose malaria in comparison to comatose individuals [54]; c) the association of high anti-*P. falciparum *IgE levels with a reduced risk of developing clinical malaria [55,56].

On the other hand, other evidence suggests that IgE could play a role in the pathogenesis of malaria; for example: a) there is an increase of the levels of IgE among individuals suffering from severe malaria in comparison to uncomplicated malaria [17,57-59]; b) IgE deposits were found in brain microvessels and on parasited erythrocytes from cerebral malaria patients [19] and in placentas infected with *P. falciparum *[20]. Immunocomplexes of IgE with antigen or with IgG anti-IgE could induce the expression of Fcε receptors in monocytes and the interaction with these receptors leads to the cellular activation and liberation of TNF [60,61]. Although TNF may protect against the parasites, local superproduction leads to tissue damage; in addition, elevated levels in the blood are related to sickness and death in cases of severe malaria [62,63]. Here, higher levels of IgE were observed among subjects suffered from complicated malaria, though the difference was not significantly higher (*P *= 0.07) for specific IgE found in the group of individuals with complicated malaria.

The frequencies of positivity and the levels of the IgM antibodies were higher among the primo-infected individuals than among those who claimed to have had previous bouts of malaria, decreasing with each additional previous episode. Primo-infected individuals were expected to have high IgM antibody levels, since IgM is the first class of antibody produced as a primary humoral response. This must also contribute to the predominance of IgM among subjects with complicated forms of malaria, since the majority of the primo-infected subjects were suffering from such forms of the disease.

The frequency of positivity of IgA antibodies does not have a correlation with the number of previous malaria episodes, though the IgA levels decreased with each previous episode. These results were in line with previous results when the predominance of IgA antibodies among primo-infected subjects was observed Since IgA deficiency has been observed among certain populations [64], total IgA levels were also investigated in this population, but these levels were normal.

## Conclusion

The selection of the isotype and the antibody affinity maturation of an antibody are the results of a process of differentiating B cells that occurs in the germinative centers of the lymph nodes of an immune individual. However, functional affinity or avidity depends on the antigen and on the selection of cell clones that produce antibodies with better cynetics for binding to the epitope. The selection of isotypes is a complex phenomenon that depends on several different mechanisms, many of which are still not fully understood [65]. The gradual development of protective immunity against malaria probably corresponds to a progressive modification in the regulation of the immune response and not to the slow development of a response or to poorly immunogenic or highly polymorphic molecules, as was believed for some time. Acquiring immunity would appear to be related to the ability to develop antibodies that, when bound to the antigen, are capable of activating cells by setting off defense mechanisms, reducing the proportion of antibodies with the same specificity and blocking cell activation [6]. Immunity to malaria results from a strong humoral immune response against multiple antigenic targets, as suggested by the inverse association of the risk of malaria with the breadth specificity for distinct merozoite antigens, AMA1, MSP-2, and MSP-3 [66], so it would be of interest to investigate further the roles of different isotypes against multiple vaccine candidate antigens in different populations.

In conclusion, the work reported here suggests a differential regulation in the anti-*P. falciparum *antibody profile in different clinical expressions of malaria. Interactions of the antibodies IgG1, IgG2 and IgG3 were predominant among individuals from the AS and UM groups, while interactions between IgG4 and IgE prevailed among individuals from the CM group. The IgG2/IgG4 levels were higher in the AS and UM groups. The IgE/IgG levels were significantly higher among CM individuals than among those without clinical complications, which may suggest that the IgG and IgE antibodies reactive against the same epitopes could play exactly opposing functions during the infection. Therefore, the relationship between the antibodies IgG/IgE, IgG1-IgG2-IgG3/IgG4, IgG2/IgG4 and IgG4-IgE/IgG2 could reflect the balance between protection and pathogenesis in malaria.

These results show that even in populations from regions with unstable malaria transmission, a certain degree of naturally acquired immunity can develop when the right antibodies are produced. Also, the findings can contribute to a better understanding of the role of different isotypes in immunity to malaria and may provide new insights into the development of malaria control strategies, especially vaccination.

## Competing interests

The authors declare that they have no competing interests.

## Authors' contributions

FMSL made technical contributions to the entire work, including data interpretation of data and manuscript drafting. RRD, MVL and MGA contributed to patient selection and follow-up. AWF contributed to the conception of the study. MCAS assisted in the statistical analysis, interpretation of data and manuscript drafting. SLM participated in the conception, design, analysis and interpretation of data, and the final approval of the version to be published.
